# Romosozumab Versus Teriparatide for Fracture Prevention, Cardiovascular Events, and Mortality in Osteoporosis: A Propensity Score-Matched Real-World Cohort Study

**DOI:** 10.3390/diagnostics16152375

**Published:** 2026-07-28

**Authors:** Jer-Yung Chen, Shih-Hung Tsai, Wen-Tien Wu, Cheng-Huan Peng, Ting-Kuo Yao, Ru-Ping Lee, Ji-Ze Hsu, Jen-Hung Wang, Chen-Pei Ho, Kuang-Ting Yeh

**Affiliations:** 1 School of Medicine, Tzu Chi University, Hualien 970374, Taiwan; 111311147@gms.tcu.edu.tw (J.-Y.C.); 111311114@gms.tcu.edu.tw (S.-H.T.); timwu@tzuchi.com.tw (W.-T.W.); peng0913@tzuchi.com.tw (C.-H.P.); 2 Department of Orthopedics, Hualien Tzu Chi Hospital, Buddhist Tzu Chi Medical Foundation, Hualien 970473, Taiwan; tayao0318@tzuchi.com.tw; 3 Institute of Medical Sciences, Tzu Chi University, Hualien 970374, Taiwan; fish@gms.tcu.edu.tw; 4 Department of Medical Research, Hualien Tzu Chi Hospital, Buddhist Tzu Chi Medical Foundation, Hualien 970473, Taiwan; jize930@gmail.com (J.-Z.H.); paulwang@tzuchi.com.tw (J.-H.W.); 5 Department of Pharmacy, Hualien Tzu Chi Hospital, Buddhist Tzu Chi Medical Foundation, Hualien 970473, Taiwan; 6 School of Nursing, College of Nursing, Tzu Chi University, Hualien 970302, Taiwan; 7 Graduate Institute of Clinical Pharmacy, Tzu Chi University, Hualien 970374, Taiwan; 8 Department of Medical Education, Hualien Tzu Chi Hospital, Buddhist Tzu Chi Medical Foundation, Hualien 970473, Taiwan

**Keywords:** romosozumab, teriparatide, osteoporotic fracture, propensity score matching, real-world evidence

## Abstract

**Background**: Romosozumab and teriparatide are approved osteoanabolic therapies for osteoporosis, but direct real-world comparisons remain limited. In this study, we evaluated comparative effectiveness and selected safety outcomes. **Methods**: Female patients with osteoporosis aged ≥ 50 years who initiated romosozumab or teriparatide were identified in the TriNetX Global Network from January 2020 through December 2025. Outcomes included any osteoporotic fracture, spine or hip fracture, major adverse cardiovascular events (MACE; a nonfatal composite of acute myocardial infarction and stroke), and all-cause mortality, which was analyzed separately. Propensity score matching balanced measured baseline characteristics. Hazard ratios (HRs) and 95% confidence intervals (CIs) were estimated using Cox regression. **Results**: After 1:1 propensity score matching, each matched cohort included 8616 patients, with follow-up administratively limited to 3 years. Romosozumab initiation was associated with lower risks of any osteoporotic fracture (445 vs. 832 events; HR, 0.68; 95% CI, 0.61–0.77) and spine or hip fracture (341 vs. 682 events; HR, 0.63; 95% CI, 0.55–0.72). The primary analysis also showed a lower estimated risk of MACE (145 vs. 255 events; HR, 0.80; 95% CI, 0.65–0.98), but this association was not consistent across sensitivity analyses. All-cause mortality did not differ significantly (81 vs. 126 events; HR, 0.96; 95% CI, 0.72–1.27). **Conclusions**: Romosozumab initiation was associated with lower fracture incidence than teriparatide initiation. The cardiovascular and mortality findings do not establish superiority, equivalence, or cardioprotection and should be interpreted cautiously.

## 1. Introduction

Osteoporosis is a systemic skeletal disorder characterized by reduced bone mass, deterioration of bone microarchitecture, and an increased risk of fragility fractures. Osteoporosis has emerged as a major global public health challenge in the context of population aging [1]. More than 200 million individuals worldwide are affected, including approximately 30% of postmenopausal women aged ≥50 years. Both prevalence and associated healthcare costs are projected to double by 2050 [2]. Osteoporotic fractures, particularly those of the hip and vertebrae, represent the principal clinical consequence of osteoporosis. From an orthopedic perspective, osteoporotic fractures, particularly vertebral compression fractures and hip fractures, represent a major clinical challenge because they are associated with increased mortality, long-term disability, and a substantial surgical burden. Thus, they impose a substantial burden on patients, healthcare systems, and societies, with direct fracture-related costs in the European Union estimated at €32 billion in 2000 [2,3].

From an orthopedic perspective, vertebral compression fractures and hip fractures commonly necessitate surgical intervention. Perioperative bone quality directly influences implant stability and surgical outcomes, underscoring the importance of optimizing pharmacological management prior to and following orthopedic procedures. However, despite the availability of effective therapies, fewer than 30% of patients receive guideline-concordant treatment following fragility fractures, highlighting a substantial care gap, particularly among older high-risk populations [1,4]. This burden is expected to further increase in the Asia–Pacific region [5].

The development of romosozumab was informed by genetic studies of rare bone disorders with extreme skeletal phenotypes. Sclerosteosis is a condition characterized by excessive bone formation, and loss-of-function mutations in *SOST* were first identified as the cause. *SOST* encodes sclerostin, thereby establishing sclerostin as a key negative regulator of bone mass [6]. This role is further supported by markedly increased bone density and strength in *SOST* knockout mouse models [7]. Sclerostin is primarily secreted by osteocytes. Mechanistically, it inhibits osteoblastic bone formation through antagonism of canonical Wnt signaling by binding to the LRP5/6 co-receptors [8]. These findings provided the biological rationale for the development of romosozumab, a monoclonal antibody targeting sclerostin. Subsequent clinical trials have demonstrated that romosozumab can increase bone mineral density (BMD) and reduce fracture risk [8,9].

In particular, a comprehensive clinical development program, including phase II dose-finding and phase III pivotal trials, has established the efficacy and safety of romosozumab as a first-in-class agent with the dual effects of stimulating bone formation while inhibiting bone resorption. In the phase III FRAME trial (*n* = 7180 postmenopausal women with osteoporosis), monthly subcutaneous romosozumab (210 mg) administered for 12 months reduced the risk of new vertebral fractures by 73% and clinical fractures by 36%. These benefits were sustained following sequential denosumab therapy, with a 75% relative reduction in vertebral fractures at 24 months [10]. The phase III ARCH trial in high-risk populations demonstrated that 12-month romosozumab followed by alendronate significantly reduced the risk of new vertebral fractures by 48%, clinical fractures by 27%, nonvertebral fractures by 19%, and hip fractures by 38% [11]. A dose-ranging study has also shown that the 210-mg monthly regimen achieves substantial BMD gains, including an 11.3% increase in lumbar spine BMD at 12 months [9]. Similar efficacy has been observed across populations, including postmenopausal Japanese women and men with osteoporosis [12,13].

Among available osteoanabolic therapies, teriparatide remains a widely used agent and is therefore a clinically relevant comparator for romosozumab. Although both therapies stimulate bone formation, their mechanisms differ. Teriparatide increases bone formation and resorption, whereas romosozumab promotes bone formation while inhibiting resorption. Head-to-head studies and meta-analyses have shown greater gains in spine and hip BMD with romosozumab, including advantages in cortical bone [14,15]. This difference may be relevant after long-term bisphosphonate therapy, when the anabolic response to teriparatide can be attenuated [14]. Both agents reduce vertebral fracture risk, whereas the ARCH trial demonstrated hip-fracture reduction with a romosozumab-to-alendronate sequence [11]. Their administration also differs: teriparatide is generally self-injected daily, whereas romosozumab is administered monthly by a healthcare professional. Although monthly administration may reduce treatment burden, comparative adherence was not measured in the present study.

Despite these findings, several important evidence gaps remain. First, much of the available evidence is based on short-term changes in BMD, a surrogate outcome, rather than long-term fracture endpoints. Second, data on sustained fracture prevention beyond the duration of clinical trials remain limited. Third, direct comparative effectiveness data between romosozumab and teriparatide in real-world clinical settings are scarce, particularly in diverse patient populations, including those with concurrent glucocorticoid use or cardiovascular comorbidities commonly encountered in orthopedic practice. Thus, in this study, we aimed to compare the risk of osteoporotic fractures, major adverse cardiovascular events (MACEs), and all-cause mortality between patients initiating romosozumab and those initiating teriparatide.

## 2. Materials and Methods

This retrospective study utilized data from the TriNetX Global Network (Cambridge, MA, USA), which includes more than 150 healthcare organizations and over 200 million patients. All data within TriNetX are de-identified and derived from electronic health records (EHRs) contributed by participating institutions, including demographics, diagnoses (International Classification of Diseases, Tenth Revision, Clinical Modification [ICD-10-CM] codes), medication prescriptions (Anatomical Therapeutic Chemical [ATC] codes), procedures, laboratory values, and mortality information. All data collection, processing, and transmission procedures adhered to applicable data protection regulations governing participating institutions [16,17]. The study protocol was reviewed and approved by the Research Ethics Committee of Hualien Tzu Chi Hospital (IRB112-133-C; approval date: 9 June 2023). The requirement for informed consent was waived because of the retrospective design and the exclusive use of de-identified data, which posed minimal risk to participants.

**Figure 1:**
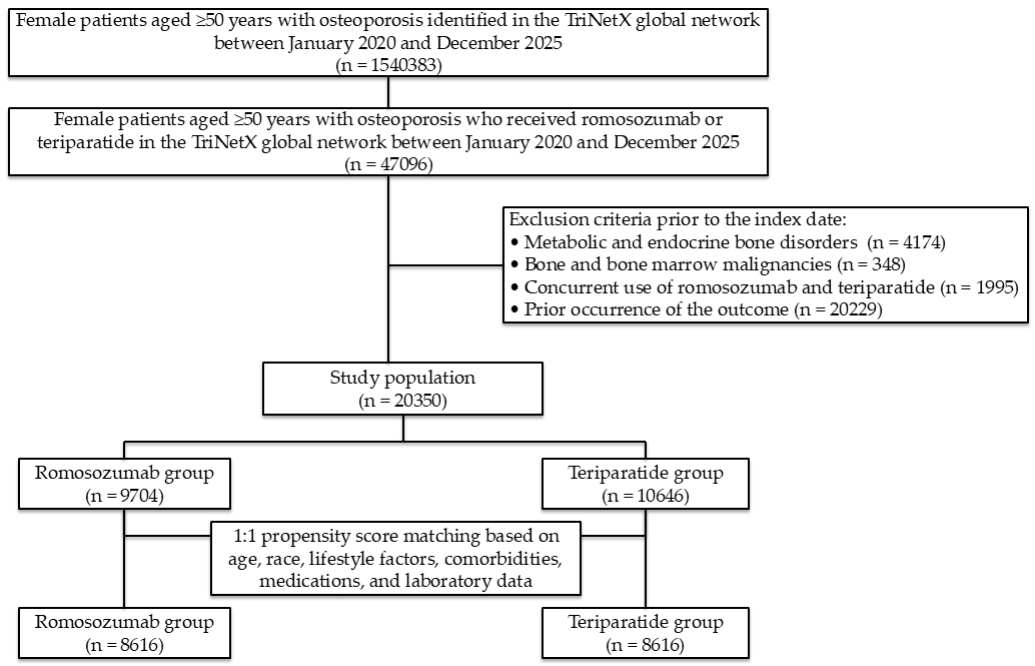
Flowchart of study population selection from the TriNetX global network.

This study included female patients aged ≥ 50 years with osteoporosis (ICD-10-CM code M81) identified in the TriNetX Global Network between January 2020 and December 2025. Eligible patients had a record of romosozumab (ATC code M05BX06) or teriparatide (ATC code H05AA02) during the study period. To implement an active-comparator new-user design, patients were required to have no record of either romosozumab or teriparatide during the 1-year baseline period before the index date. The index date was the date of that first qualifying prescription record. Treatment assignment was fixed on the index date, and no post-index exposure qualification period, minimum treatment duration, subsequent prescription, or survival requirement was used to define cohort eligibility. Patients with records of both study drugs on the index date were excluded. Subsequent use of the comparator drug during follow-up did not alter the original treatment assignment in the primary analysis and was addressed in the no-treatment-switching sensitivity analysis. Patients with metabolic or endocrine bone disorders, bone or bone marrow malignancies, or occurrence of the outcome under evaluation before the index date were also excluded. These criteria were applied to improve attribution to the index therapy and identify incident outcomes. Figure 1 summarizes cohort selection.

Outcome follow-up began on the day after the index date for both treatment groups. The primary outcome was the first occurrence of a new osteoporotic fracture, identified using ICD-10-CM codes for hip fracture (S72), vertebral fracture (M48.4, M48.5, S22, and S32), distal forearm or wrist fracture (S52), and proximal humerus fracture (S42). Secondary outcomes were: (1) spine or hip fracture; (2) major adverse cardiovascular events (MACE), defined as a nonfatal composite of acute myocardial infarction (I21–I22) and ischemic or hemorrhagic stroke (I60–I63); and (3) all-cause mortality. All-cause mortality was not included in the MACE composite and was analyzed independently. For each outcome, patients were followed from the day after the index date until the earliest occurrence of that outcome, loss to follow-up (the last documented EHR contact), 31 December 2025, or administrative censoring 3 years after the index date.

Baseline covariates were assessed during the 1-year period before the index date. The propensity score model included all variables reported in Table 1: age; race; nicotine dependence; alcohol-related disorders; diabetes mellitus; hyperlipidemia; hypertension; chronic kidney disease; neoplasms; hypothyroidism; hyperthyroidism; inflammatory polyarthropathies; glucocorticoids; calcium; vitamin D and analogues; bisphosphonates; denosumab; aromatase inhibitors; obesity (BMI ≥ 30 kg/m^2^); total cholesterol ≥ 200 mg/dL; and hemoglobin A1c ≥ 6.5%. Age was modeled as a continuous variable, whereas the remaining covariates were represented as categorical or binary indicators. For BMI, total cholesterol, and hemoglobin A1c, measurements meeting or exceeding the prespecified thresholds were coded as indicating the corresponding abnormality. Values below the threshold and the absence of a recorded measurement were both coded as no documented abnormality. No statistical imputation was performed. Supplementary Table S1 summarizes the missingness of the underlying BMI, total cholesterol, and hemoglobin A1c measurements before matching.

Propensity score estimation, matching, balance assessment, survival analysis, and sensitivity analyses are described in the Statistical Analysis section.

### Statistical Analysis

To reduce confounding by indication and other measured baseline differences between the treatment groups, propensity scores for assignment to romosozumab rather than teriparatide were estimated within the TriNetX analytics platform using logistic regression based on the prespecified baseline covariates listed above. Patients were matched 1:1 using greedy nearest-neighbor matching without replacement, with a caliper width equal to 0.1 of the pooled standard deviation of the propensity score. Covariate balance before and after matching was assessed using absolute standardized mean differences (SMDs), with an SMD < 0.10 considered indicative of adequate balance [18,19]. Absolute SMDs before and after matching are presented in a Love plot (Supplementary Figure S1).

Event-free survival was estimated using the Kaplan–Meier method, and time-to-event distributions were compared using log-rank tests. Cumulative event probabilities were presented as one minus the Kaplan–Meier estimate. For nonfatal outcomes, death was treated as a censoring event; accordingly, these estimates are not competing-risk cumulative incidence functions. Cox proportional hazards models were used to estimate hazard ratios (HRs) and 95% confidence intervals (CIs). The proportional hazards assumption was evaluated using the platform-based Schoenfeld residual diagnostic. Prespecified subgroup analyses were conducted according to age (50–64 vs. ≥ 65 years), glucocorticoid use, and hypertension. Because formal treatment-by-subgroup interaction tests were not available, subgroup analyses were considered descriptive and were not interpreted as evidence of effect modification.

Three sensitivity analyses were conducted. First, a no-treatment-switching (“clean-drug”) analysis excluded patients with a record of the comparator agent during follow-up. Because this restriction was based on post-index information, the analysis was interpreted as a supportive, per-protocol-like sensitivity analysis. Second, a 6-month landmark analysis excluded events occurring during the first 6 months and began follow-up at month 6 among patients who remained under observation and outcome-free, applying identical criteria to both treatment groups. Third, E-values were calculated to quantify the minimum strength of association, on the risk-ratio scale, that an unmeasured confounder would need to have with both treatment assignment and the outcome to explain the observed association [20].

Cohort construction, propensity score matching, balance assessment, outcome analyses, and sensitivity analyses were performed within the TriNetX analytics platform. Aggregate outputs from TriNetX were used to generate the Kaplan–Meier curves and Love plot using R version 4.5.1. Because TriNetX does not permit the export of individual-level event and censoring times, restricted mean survival time analyses, time-varying Cox models, and exact interval-specific risk-set counts could not be calculated externally. All statistical tests were two-sided, and a *p*-value < 0.05 was considered statistically significant. Because no adjustment for multiple comparisons was performed, secondary outcome, subgroup, and sensitivity analyses were interpreted as supportive or exploratory.

## 3. Results

Table 1 presents the baseline characteristics before and after propensity score matching. Before matching, 9704 romosozumab users and 10,646 teriparatide users were included. Romosozumab users were older than teriparatide users (69.0 ± 7.9 vs. 66.9 ± 8.3 years; SMD = 0.250), and several other baseline covariates were imbalanced. After 1:1 matching, each group included 8616 patients. All covariates included in the propensity score model achieved adequate balance, with absolute SMDs < 0.10 and a maximum post-matching SMD of 0.014 (Supplementary Figure S1). The median observed follow-up duration was 478 days (IQR, 694 days) in the romosozumab group and 1095 days (IQR, 655 days) in the teriparatide group. Before matching, baseline data were missing for BMI in 36.6% and 33.9%, total cholesterol in 63.2% and 67.9%, and hemoglobin A1c in 75.6% and 78.9% of the romosozumab and teriparatide groups, respectively (Supplementary Table S1).

**Figure 2:**
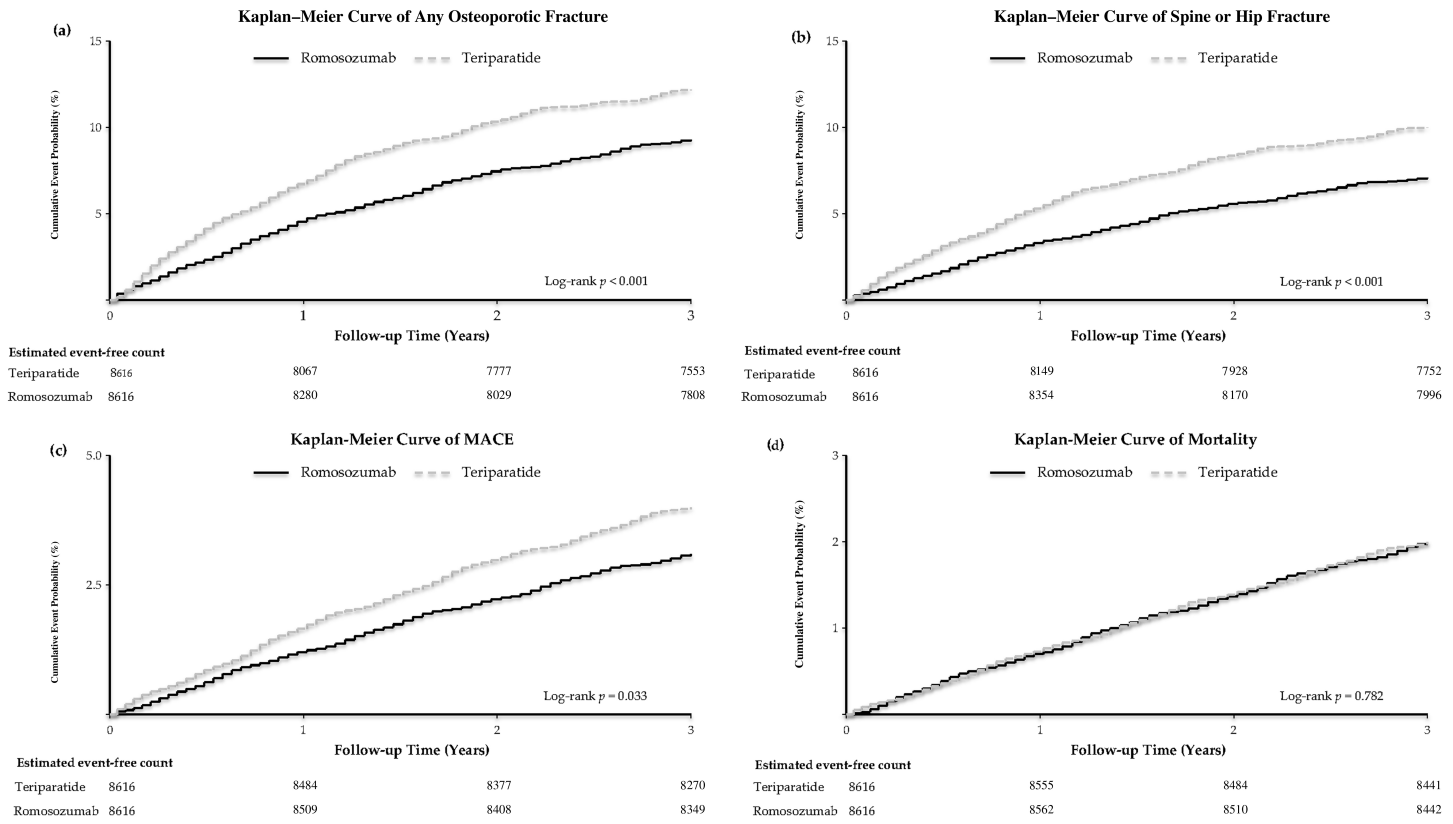
Kaplan–Meier-derived cumulative event probabilities for clinical outcomes in patients treated with romosozumab versus teriparatide after propensity score matching. (**a**) any osteoporotic fracture; (**b**) spine or hip fracture; (**c**) MACE; (**d**) mortality. The curves display one minus the Kaplan–Meier estimate. For nonfatal outcomes, death was treated as a censoring event; therefore, these curves are not competing-risk cumulative incidence functions. Estimated event-free counts are presented beneath each curve. Because individual-level time-to-event data and exact risk-set counts were unavailable, the displayed counts were approximated using aggregate survival probabilities provided by the TriNetX platform. MACE: major adverse cardiovascular events.

Figure 2 shows Kaplan–Meier estimates after propensity score matching. The approximate 1-year and 3-year Kaplan–Meier-derived cumulative event probabilities for any osteoporotic fracture were 3.9% and 9.4%, respectively, in the romosozumab group versus 6.4% and 12.3% in the teriparatide group; for spine or hip fracture, the corresponding probabilities were 3.0% and 7.2% versus 5.4% and 10.0% (log-rank *p* < 0.001 for both outcomes). For MACE, the corresponding probabilities were 1.2% and 3.1% versus 1.5% and 4.0% (log-rank *p* = 0.033); for mortality, they were 0.6% and 2.0% versus 0.7% and 2.0% (log-rank *p* = 0.782). These probabilities represent one minus the Kaplan–Meier estimate. For nonfatal outcomes, death was censored; therefore, the estimates are not competing-risk cumulative incidence functions. The MACE result was interpreted in conjunction with the sensitivity analyses rather than as evidence of cardiovascular benefit.

Table 2 presents comparative outcome risks after propensity score matching. Compared with teriparatide initiation, romosozumab initiation was associated with lower risks of any osteoporotic fracture (445 vs. 832 events; HR, 0.68; 95% CI, 0.61–0.77), spine or hip fracture (341 vs. 682 events; HR, 0.63; 95% CI, 0.55–0.72), and MACE (145 vs. 255 events; HR, 0.80; 95% CI, 0.65–0.98). All-cause mortality did not differ significantly (81 vs. 126 events; HR, 0.96; 95% CI, 0.72–1.27).

Table 3 presents prespecified subgroup analyses. In patients aged 50–64 years, romosozumab initiation was associated with lower risks of any osteoporotic fracture (HR, 0.75; 95% CI, 0.59–0.95) and spine or hip fracture (HR, 0.71; 95% CI, 0.54–0.93), whereas MACE did not differ significantly. Corresponding fracture associations were observed in patients aged ≥ 65 years (HR, 0.70 and 0.64, respectively), glucocorticoid users (HR, 0.66 and 0.65), and patients with hypertension (HR, 0.65 and 0.63). MACE and mortality estimates in these strata did not show statistically significant between-group differences. These stratum-specific estimates are descriptive. Formal treatment-by-subgroup interaction tests were not available, and between-stratum differences should not be interpreted as evidence of effect modification.

Table 4 presents the sensitivity analyses. In the no-treatment-switching analysis, romosozumab initiation remained associated with lower risks of any osteoporotic fracture (HR, 0.70; 95% CI, 0.62–0.79) and spine or hip fracture (HR, 0.66; 95% CI, 0.58–0.75), whereas MACE and mortality did not differ significantly. In the 6-month landmark analysis, the HRs were 0.72 (95% CI, 0.61–0.85) for any osteoporotic fracture, 0.69 (95% CI, 0.57–0.84) for spine or hip fracture, 0.74 (95% CI, 0.57–0.97) for MACE, and 0.95 (95% CI, 0.67–1.36) for mortality. E-values for the point estimates were 2.30, 2.55, 1.81, and 1.25, respectively.

## 4. Discussion

In this real-world study of 8616 matched pairs, romosozumab initiation was associated with lower average hazards of any osteoporotic fracture (HR, 0.68; 95% CI, 0.61–0.77) and spine or hip fracture (HR, 0.63; 95% CI, 0.55–0.72) than teriparatide initiation. These associations persisted in the no-treatment-switching and 6-month landmark analyses. Stratum-specific subgroup estimates were generally in the same direction, but no formal interaction tests were performed; therefore, the subgroup findings should not be interpreted as evidence of effect modification. By contrast, the lower MACE estimate in the primary analysis was not maintained in every sensitivity analysis, and mortality did not differ significantly. Thus, the fracture findings were more consistent than the cardiovascular or mortality findings.

The fracture association is biologically plausible. Romosozumab inhibits sclerostin and enhances canonical Wnt signaling, increasing bone formation while reducing bone resorption [8]. Teriparatide stimulates bone formation but also increases remodeling and resorption [14]. These mechanistic differences may influence trabecular and cortical strength. The numerically stronger association for spine or hip fracture than for the broader fracture composite is also compatible with randomized evidence from the ARCH trial, in which a romosozumab-to-alendronate sequence reduced hip-fracture risk [11]. Nevertheless, the present observational comparison evaluates treatment initiation and cannot establish that mechanism alone caused the observed difference.

The fracture estimates changed little in the no-treatment-switching analysis (HR, 0.70 for any osteoporotic fracture; HR, 0.66 for spine or hip fracture). This supports the primary association but does not eliminate selection bias, because exclusion based on post-index switching may select patients with different persistence, tolerability, or clinical trajectories.

The 6-month landmark analysis yielded HRs of 0.72 for any osteoporotic fracture and 0.69 for spine or hip fracture. Excluding early events reduces the influence of fractures that may primarily reflect skeletal fragility already present at treatment initiation. However, landmarking also conditions on remaining observed and event-free for 6 months; these estimates should therefore be viewed as complementary rather than as replacements for the primary analysis.

For MACE, significance was observed in the primary and 6-month landmark analyses but not in the no-treatment-switching analysis. This inconsistency, together with the cardiovascular signal reported in earlier randomized trials [10,11], argues against interpreting the present estimates as evidence of cardioprotection or cardiovascular equivalence. They instead indicate that no consistent excess or benefit was demonstrated relative to teriparatide within the available follow-up. Dedicated studies with prespecified cardiovascular endpoints and adequate event counts remain necessary.

Our findings add to the increasing real-world evidence on osteoanabolic therapy for osteoporosis, particularly that derived from federated electronic health record networks. Lu et al. reported that, after propensity score matching, romosozumab was associated with a lower risk of osteoporotic fractures than PTH receptor agonists (HR, 0.711; 95% CI, 0.542–0.931). The associations were particularly evident among women, patients aged ≥ 65 years, and those with prior fractures [21]. The present study contributes complementary and methodologically refined evidence by focusing specifically on teriparatide as a single active comparator; incorporating both U.S. and Asia–Pacific patient populations from the TriNetX platform; and jointly evaluating fracture, cardiovascular, and mortality endpoints within a unified analytic framework that includes clean-drug, 6-month lag, and E-value analyses.

Cardiovascular risk remains central to romosozumab selection because serious cardiovascular events were more frequent with romosozumab than with alendronate in ARCH, although the same pattern was not observed in FRAME [10,11]. In the present study, the primary MACE estimates favored romosozumab, but the association was attenuated in the no-treatment-switching analysis. Recent TriNetX evidence has likewise reported fewer 3-point MACEs with romosozumab than with parathyroid hormone analogues [22], but differences in comparators, outcome definitions, and follow-up limit direct comparison. Our findings should not be construed as demonstrating cardiovascular superiority, equivalence, or absence of risk. Individual cardiovascular risk assessment remains essential, particularly for patients with established cardiovascular disease or multiple risk factors.

All-cause mortality did not significantly differ between the treatment groups in the present study. This finding warrants careful interpretation in the context of the broader observational literature. Some prior real-world analyses have reported lower mortality among romosozumab users than among teriparatide users. Tsai et al. reported an HR of 0.68 (95% CI, 0.49–0.94) in a TriNetX-based cohort [23]. Several methodological factors, including population eligibility criteria, age thresholds, exclusion criteria related to prior cerebrovascular disease, study period, outcome hierarchy, and follow-up structure, may account for differences across studies. The relatively low number of deaths observed in our matched cohort may also have constrained statistical precision for the mortality comparison. In addition, the 3-year administrative censoring window may have provided insufficient time for long-emerging differences in mortality to manifest. Importantly, the absence of a significant mortality difference in both the primary and clean-drug analyses reduces the likelihood that differential mortality or depletion of susceptible patients fully accounts for the lower fracture incidence observed among romosozumab initiators. Nevertheless, because death remains a competing event for fracture outcomes, future studies incorporating competing-risk methods may further clarify the extent to which mortality affects fracture risk estimation [24].

The fracture findings may inform multidisciplinary osteoporosis care relevant to orthopedic practice, because vertebral and hip fractures frequently require surgical treatment and are associated with disability and mortality. Within the older-patient and glucocorticoid-user strata, fracture estimates were lower, but these descriptive findings do not establish that treatment effects differed across subgroups. However, in this study, we did not measure BMD response, implant fixation, fusion, perioperative complications, functional recovery, or other surgical outcomes. Therefore, the results support consideration of comparative fracture risk when selecting osteoanabolic therapy, but they do not establish that romosozumab improves operative outcomes or should be routinely preferred for perioperative bone optimization. Such questions require prospective studies designed around orthopedic endpoints.

E-value analyses provide additional context regarding the potential influence of unmeasured confounding [20]. For the primary osteoporotic fracture outcome, the E-value was 2.30 for the point estimate and was 1.92 for the confidence limit closest to the null. This indicated that a moderately strong unmeasured confounder would be required to fully explain away the observed fracture association. For spine or hip fracture, the corresponding E-values were 2.55 and 2.12, reflecting comparatively greater robustness for this clinically important outcome. E-values in the glucocorticoid user subgroup were comparable to those in the overall cohort (2.40 and 2.45, respectively), suggesting consistent robustness across this high-risk population. In contrast, E-values for MACE and all-cause mortality were smaller (1.81 and 1.25 for the point estimates, respectively), indicating that the safety findings were more susceptible to potential unmeasured confounding and should be interpreted with greater caution.

The proportional hazards assumption was not met for any osteoporotic fracture (Schoenfeld residual *p* = 0.0012) or spine or hip fracture (*p* < 0.001) [25]. The reported Cox estimates should therefore be interpreted as average relative hazards over follow-up, not as constant treatment effects. Nonproportionality is plausible because romosozumab is limited to 12 months and is usually followed by antiresorptive therapy [26], whereas subsequent treatment patterns can alter later fracture risk. The aggregate TriNetX output available for this analysis did not permit a validated time-varying Cox model or calculation of restricted mean event-free time. Kaplan–Meier curves were retained to show the cumulative event-probability pattern, and future studies with patient-level event and censoring times should report time-varying effects, restricted mean survival time differences, or landmark-specific estimates.

This study has several strengths. The active-comparator initiator design provides a clinically relevant head-to-head comparison, and the large TriNetX network enabled analysis across diverse real-world settings. Matching achieved excellent balance in recorded covariates, as documented numerically and in Supplementary Figure S1. Fracture associations persisted across complementary no-treatment-switching, landmark, and E-value analyses. Subgroup estimates were descriptive because formal interaction tests were unavailable. Formal assessment of the proportional hazards assumption also enabled appropriately qualified interpretation of Cox estimates.

Some limitations merit emphasis. First, residual confounding remains possible despite matching. BMD and T-scores, FRAX estimates, osteoporosis severity, frailty, falls, serum 25-hydroxyvitamin D, physical function, socioeconomic factors, and prescriber preference were unavailable or incompletely recorded. Missingness of the underlying laboratory measurements was substantial, particularly for cholesterol and hemoglobin A1c. Because a normal value and an absent test record were both coded as no documented abnormality, patients with genuinely normal findings could not be distinguished from those who were not tested; balance in the derived binary indicators therefore does not ensure complete laboratory comparability. Given the high degree of missingness, particularly for cholesterol and hemoglobin A1c, apparent balance may reflect similar patterns of absent testing rather than true biomarker comparability; misclassification and residual confounding may therefore be substantial. Channeling of a newer, more costly therapy according to access, disease severity, or clinician judgment may also remain. E-values quantify the strength an unmeasured confounder would require to explain an association but do not remove confounding, account for multiple correlated unmeasured factors, or correct measurement error.

Second, exposure and outcomes were identified from EHR prescription, medication, and diagnosis records rather than through protocolized adjudication. The TriNetX analytics platform does not provide sufficiently detailed information on prescription dose, treatment duration, refill behavior, actual administration, or temporal changes in medication use. Therefore, treatment adherence, persistence, and dose completion could not be reliably assessed. This limitation is particularly relevant because teriparatide requires daily self-injection, whereas romosozumab is typically administered monthly by healthcare professionals; differential adherence or persistence could therefore contribute to the observed difference in effectiveness. Fractures not captured within participating healthcare systems or not appropriately coded may also have been missed. Third, the proportional hazards assumption was violated for fracture outcomes, limiting Cox estimates to average relative hazards over follow-up; RMST and time-varying Cox analyses were unavailable within TriNetX. Fourth, median follow-up was shorter for romosozumab than for teriparatide (478 vs. 1095 days), potentially reflecting the more recent uptake of romosozumab. Although Cox and Kaplan–Meier methods accommodate right censoring under an independent-censoring assumption, differential follow-up may reduce the precision and robustness of later estimates. Exact interval-specific risk-set counts were not available. Fifth, cardiovascular events and deaths were secondary outcomes with relatively few events. Sixth, post-romosozumab antiresorptive treatment was not comprehensively characterized; differences in subsequent denosumab or bisphosphonate use could preserve, attenuate, or confound longer-term associations, and outcomes beyond 12 months cannot be attributed solely to the index drug. Finally, findings from participating organizations may not generalize to all populations or healthcare systems.

## 5. Conclusions

In this large propensity score-matched cohort, romosozumab initiation was associated with lower incidences of any osteoporotic fracture and spine or hip fracture than teriparatide initiation, and the fracture associations persisted across the prespecified sensitivity analyses. All-cause mortality did not differ significantly. Because MACE estimates varied across sensitivity analyses and residual confounding, differential adherence, sequential therapy, and nonproportional hazards remain possible, the cardiovascular findings should not be interpreted as demonstrating superiority, equivalence, or cardioprotection. The results may inform shared osteoanabolic treatment decisions for patients at high fracture risk, including those encountered in orthopedic practice, but do not directly establish benefits for surgical outcomes. Prospective studies with patient-level follow-up, treatment-persistence data, sequential-therapy characterization, and prespecified cardiovascular and orthopedic endpoints are warranted.

## Figures and Tables

**Table 1:** Baseline characteristics of female patients with osteoporosis treated with romosozumab or teriparatide before and after propensity score matching.

Item	Before PSM		SMD	After PSM		SMD
	Romosozumab (*n* = 9704)	Teriparatide (*n* = 10,646)		Romosozumab (*n* = 8616)	Teriparatide (*n* = 8616)	
Age (years), mean ± SD	69.0 ± 7.9	66.9 ± 8.3	0.25	68.3 ± 7.7	68.4 ± 7.8	0.014
Race, *n* (%)						
White	6973 (71.9%)	8032 (75.4%)	0.082	6324 (73.4%)	6329 (73.5%)	0.001
Black or African American	212 (2.2%)	301 (2.8%)	0.041	205 (2.4%)	194 (2.3%)	0.009
Asian	1162 (12.0%)	895 (8.4%)	0.118	879 (10.2%)	853 (9.9%)	0.01
Lifestyle factors, *n* (%)						
Nicotine dependence	311 (3.2%)	446 (4.2%)	0.052	294 (3.4%)	295 (3.4%)	0.001
Alcohol related disorders	61 (0.6%)	84 (0.8%)	0.019	58 (0.7%)	58 (0.7%)	0.001
Comorbidities, *n* (%)						
Diabetes mellitus	713 (7.3%)	893 (8.4%)	0.039	656 (7.6%)	643 (7.5%)	0.006
Hyperlipidemia	3281 (33.8%)	2976 (28.0%)	0.127	2681 (31.1%)	2683 (31.1%)	0.001
Hypertensive diseases	2480 (25.6%)	2719 (25.5%)	0.001	2171 (25.2%)	2169 (25.2%)	0.001
Chronic kidney disease	492 (5.1%)	399 (3.7%)	0.064	375 (4.4%)	367 (4.3%)	0.005
Neoplasms	2126 (21.9%)	1736 (16.3%)	0.143	1612 (18.7%)	1587 (18.4%)	0.008
Hypothyroidism	1328 (13.7%)	1459 (13.7%)	0.001	1168 (13.6%)	1193 (13.8%)	0.008
Hyperthyroidism	164 (1.7%)	209 (2.0%)	0.02	158 (1.8%)	151 (1.8%)	0.006
Inflammatory polyarthropathies	749 (7.7%)	1005 (9.4%)	0.062	696 (8.1%)	691 (8.0%)	0.002
Medications, *n* (%)						
Glucocorticoids	2830 (29.2%)	3350 (31.5%)	0.05	2541 (29.5%)	2558 (29.7%)	0.004
Calcium	1320 (13.6%)	1648 (15.5%)	0.053	1191 (13.8%)	1229 (14.3%)	0.013
Vitamin D and analogues	1243 (12.8%)	1803 (16.9%)	0.116	1179 (13.7%)	1203 (14.0%)	0.008
Bisphosphonates	1094 (11.3%)	1312 (12.3%)	0.033	1001 (11.6%)	1000 (11.6%)	0.001
Denosumab	701 (7.2%)	512 (4.8%)	0.102	490 (5.7%)	484 (5.6%)	0.003
Aromatase inhibitors	138 (1.4%)	39 (0.4%)	0.112	44 (0.5%)	38 (0.4%)	0.01
Laboratory, *n* (%)						
BMI ≥ 30 kg/m^2^	921 (9.5%)	1096 (10.3%)	0.027	837 (9.7%)	826 (9.6%)	0.004
Cholesterol ≥ 200 mg/dL	1680 (17.3%)	1557 (14.6%)	0.073	1386 (16.1%)	1374 (15.9%)	0.004
Hemoglobin A1c ≥ 6.5%	322 (3.3%)	398 (3.7%)	0.023	291 (3.4%)	288 (3.3%)	0.002

Abbreviations: BMI, body mass index; PSM, propensity score matching; SD, standard deviation; SMD, standardized mean difference.

**Table 2:** Comparative risk of clinical outcomes in patients treated with romosozumab versus teriparatide after propensity score matching.

Item	Romosozumab		Teriparatide		HR (95% CI)
	Event	*n*	Event	*n*	
Fracture	445	8616	832	8616	0.68 (0.61, 0.77) *
Spine or hip fracture	341	8616	682	8616	0.63 (0.55, 0.72) *
MACE	145	8616	255	8616	0.80 (0.65, 0.98) *
Mortality	81	8616	126	8616	0.96 (0.72, 1.27)

HRs were calculated with teriparatide as the reference group. An asterisk indicates statistical significance (*p* < 0.05). Abbreviations: CI, confidence interval; HR, hazard ratio; MACE, major adverse cardiovascular events.

**Table 3:** Subgroup analyses of clinical outcomes in patients treated with romosozumab versus teriparatide after propensity score matching.

Item	Fracture	Spine or Hip Fracture	MACE	Mortality
	HR (95% CI)	HR (95% CI)	HR (95% CI)	HR (95% CI)
Age group				
Age 50–64 years	0.75 (0.59, 0.95) *	0.71 (0.54, 0.93) *	0.85 (0.53, 1.37)	-
Age ≥ 65 years	0.70 (0.61, 0.80) *	0.64 (0.54, 0.74) *	0.86 (0.68, 1.08)	1.10 (0.81, 1.49)
Baseline clinical strata ^a^				
Glucocorticoid use	0.66 (0.55, 0.79) *	0.65 (0.53, 0.80) *	0.75 (0.54, 1.02)	0.94 (0.62, 1.42)
Hypertension	0.65 (0.53, 0.80) *	0.63 (0.50, 0.80) *	0.75 (0.54, 1.03)	1.31 (0.86, 2.01)

^a^ Medication use or disease status recorded during the 1-year baseline period. HRs were calculated with teriparatide as the reference group. An asterisk indicates statistical significance (*p* < 0.05). TriNetX suppresses exact counts for very small cells to protect privacy. Abbreviations: CI, confidence interval; HR, hazard ratio; MACE, major adverse cardiovascular events. Subgroup estimates are descriptive; no formal treatment-by-subgroup interaction tests were performed.

**Table 4:** Sensitivity analyses of clinical outcomes after propensity score matching.

Item	Fracture	Spine or Hip Fracture	MACE	Mortality
	HR (95% CI)	HR (95% CI)	HR (95% CI)	HR (95% CI)
No treatment switching ^a^	0.70 (0.62, 0.79) *	0.66 (0.58, 0.75) *	0.82 (0.66, 1.01)	0.98 (0.74, 1.30)
6-month landmark analysis ^b^	0.72 (0.61, 0.85) *	0.69 (0.57, 0.84) *	0.74 (0.57, 0.97) *	0.95 (0.67, 1.36)

^a^ Excluded patients with a record of the comparator drug during follow-up. ^b^ Excluded events during the first 6 months and began the at-risk period at month 6 among patients remaining observed and outcome-free. HRs were calculated with teriparatide as the reference group. An asterisk indicates statistical significance (*p* < 0.05). Abbreviations: CI, confidence interval; HR, hazard ratio; MACE, major adverse cardiovascular events.

## Data Availability

The data used in this study were accessed through the TriNetX federated analytics platform and are subject to institutional and platform restrictions. Aggregate results supporting the findings are reported in the article and Supplementary Materials; patient-level data are not available to the authors or for public release.

## Supplementary Materials

The following supporting information can be downloaded at https://www.mdpi.com/article/10.3390/diagnostics16152375/s1. Figure S1: Love plot of absolute standardized mean differences in baseline characteristics before and after 1:1 propensity score matching; Table S1: Missingness of baseline BMI and laboratory measurements before propensity score matching.

## Abbreviations

The following abbreviations are used in this manuscript:

BMD	Bone mineral density
BMI	Body mass index
CIs	Confidence intervals
EHRs	Electronic health records
HRs	Hazard ratios
ICD-10-CM	International Classification of Diseases, Tenth Revision, Clinical Modification
MACEs	Major adverse cardiovascular events
PHs	Proportional hazards
PSM	Propensity score matching
SD	Standard deviation
SMDs	Standardized mean differences
