# Supplementary material for: Romosozumab Versus Teriparatide for Fracture Prevention, Cardiovascular Events, and Mortality in Osteoporosis: A Propensity Score-Matched Real-World Cohort Study
Source: Diagnostics (Basel). 2026 Jul 28;16(15):2375. doi: 10.3390/diagnostics16152375 (PMC13464869; doi:10.3390/diagnostics16152375)
Supplement: Supplementary file 1 [file diagnostics-16-02375-s001.zip › diagnostics-4416245-supplementary.pdf]

Table S1. Missingness of baseline BMI and laboratory measurements before propensity score matching.

Measurement	Romosozumab (*n* = 9,704)	Teriparatide (*n* = 10,646)
BMI, kg/m²	3,550 (36.6%)	3,611 (33.9%)
Cholesterol, mg/dL	6,134 (63.2%)	7,231 (67.9%)
Hemoglobin A1c, %	7,341 (75.6%)	8,398 (78.9%)

Data are presented as *n* (%). Abbreviation: BMI, body mass index.

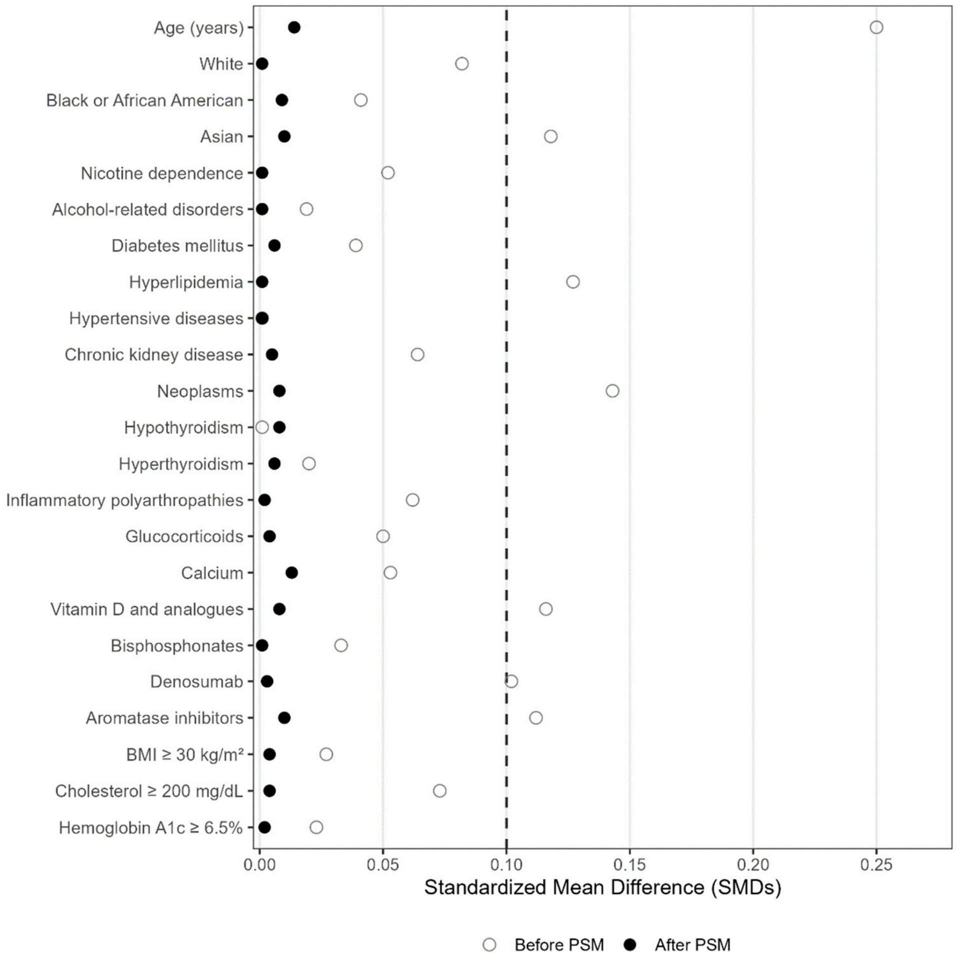

Figure S1. Love plot of absolute standardized mean differences in baseline characteristics before and after 1:1 propensity score matching. The dashed vertical line denotes the prespecified balance threshold of 0.10. All measured covariates had an absolute standardized mean difference below 0.10 after matching. Abbreviation: PSM, propensity score matching; SMD, standardized mean difference.
